# Transoral endoscopic thyroidectomy vestibular approach (TOETVA): first twelve case series in Erbil, Iraq

**DOI:** 10.25122/jml-2021-0276

**Published:** 2022-10

**Authors:** Samir Anwar Jabbar, Nooraddin Ismail Alla Werdi

**Affiliations:** 1 General Surgery Department, Erbil Teaching Hospital, Erbil, Iraq; 2 General Surgery Department, Rizgari Teaching Hospital, Hawler Medical University, Erbil, Iraq

**Keywords:** endoscopic thyroidectomy, transoral, TOETVA, scarless

## Abstract

Endoscopic thyroid surgery has gained popularity with the advances in laparoscopic and endoscopic instruments and techniques. Of all endoscopic approaches for thyroidectomy, the transoral endoscopic thyroidectomy vestibular approach (TOETVA) is the most promising procedure as it avoids neck scars and has less tissue dissection than other endoscopic techniques. This study aimed to present our first impression and initial experience with TOETVA in Erbil city. Twelve patients underwent surgery between November 2020 and April 2021, including eleven females and one male. Eleven total thyroidectomies and one lobectomy were performed. We found no significant postoperative complications. TOETVA is a safe and effective procedure, but it is challenging, needs good skills, and is a promising technique.

## INTRODUCTION

Thyroid nodularity is a common condition among thyroid diseases, being more prevalent among women. Surgical intervention is the primary intervention in managing this condition [1]. The popular surgical method is the same method that Kocher described, with some changes or modifications [2].

The development of minimally invasive procedures for the thyroid gland dates back to the mid-1990, going through many changes and trials. Many changes and modifications have been described and shifted from one procedure to another in an attempt to hide the scars or improve cosmetics in the cervical region [3].

When endoscopic thyroidectomy was invented, the procedure became popular, and many different ways were described, such as cervical endoscopy [4], axillary [5], axillo-mammary [6, 7] and retroauricular access [8], with or without robotic assistance.

The transoral endoscopic thyroidectomy by vestibular approach (TOETVA) was described by Anuwong et al. in 2016. The results were comparable to the classic open thyroidectomy but without any visible scars in the neck region [3]. What makes TOETVA different from the other approaches is the complete avoidance of neck scars. Other advantages over other endoscopic procedures for thyroidectomy are less tissue dissection and less distance to reach the thyroid gland [9]. As the gland is accessed through the vestibular approach in TOETVA, there is less manipulation of the nerves and other structures compared to other methods like mammary, axillo-mammary, retroauricular or open methods [10].

Many studies that performed TOETVA reported that the approach is very safe in preserving the recurrent laryngeal nerves [11–13]. TOETVA is no longer considered an experimental approach for some authors [14]. Several comparative studies showed favorable results of TOETVA compared to other techniques [15]. This study presents the first twelve cases in the Kurdistan Autonomous Region of Iraq.

## Material and Methods

Twelve patients were included in this study. All surgeries were performed by one surgeon (Dr. Samir A. Jabbar) at Medicano Hospital in Erbil City, the capital of the Kurdistan Region of Iraq, in cooperation with Dr. Nooraddin Ismail (Professor of General Surgery), who had a great role as a consultant to review and select patients for this procedure. Patients were informed of their surgical options, which included conventional open surgery and TOETVA. The risks and benefits of both techniques were discussed with the patients, and each patient signed informed consents.

### Inclusion criteria

- A thyroid gland diameter not larger than 10 cm or a thyroid volume not more than 45 ml;
- A benign tumor, such as a thyroid cyst, single-nodular goiter, or multinodular goiter (nodule not more than 5 cm);
- A follicular neoplasm;
- Grave's disease;
- A papillary microcarcinoma without evidence of metastasis.

### Exclusion criteria

- Patients unfit for surgery;
- Patients with previous surgery or radiation at the neck;
- A huge thyroid gland (more than 10 cm in diameter).

The following variables were taken into account: age, sex, nature of the thyroid disease, type of surgery (lobectomy or total thyroidectomy), duration of the surgery, skin bruising and other complications. The surgical technique was the same as described by Anuwong et al. [3].

### Surgical Technique

**Figure 1:**
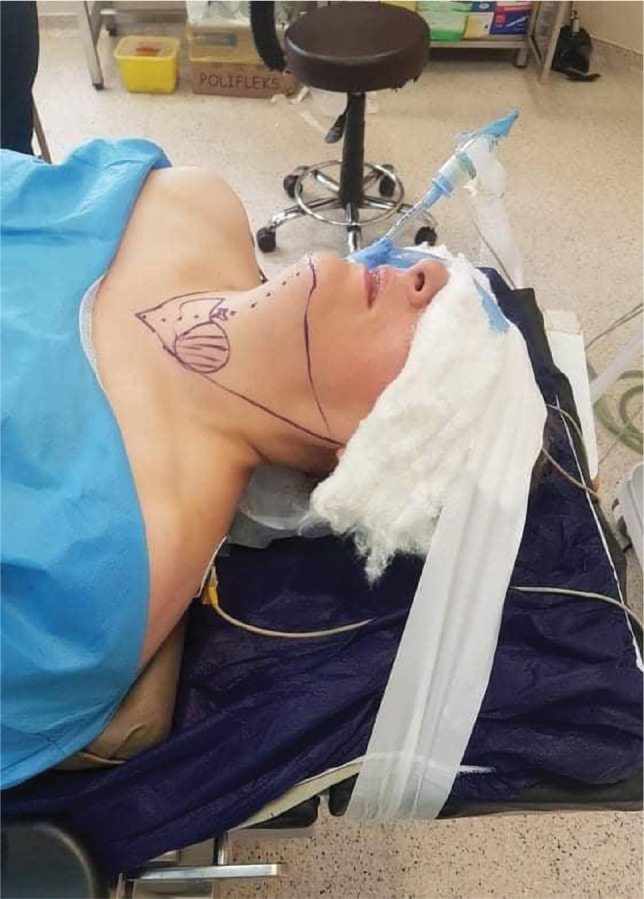
Patient's position.

Here we describe the standard technique used in TOETVA worldwide, which was first described by Anuwong et al. [3], but with some minor technical modifications in terms of positioning and preparation, like putting cotton packs over the closed eyes and fixing with plaster to protect the eyes from incidental injuries by the oral ports during manipulation. The patients were placed in a supine position, sandbags were used to raise the shoulders, and the neck was slightly extended. The head was stabilized (Figure 1). We performed all the surgeries under general anesthesia with endotracheal intubation. The neck and the lower face were prepped and draped. Chlorhexidine mouthwash was used to rinse the mouth both preoperatively and intraoperatively. In addition, a prophylactic intravenous antibiotic was used before the incision.

**Figure 2:**
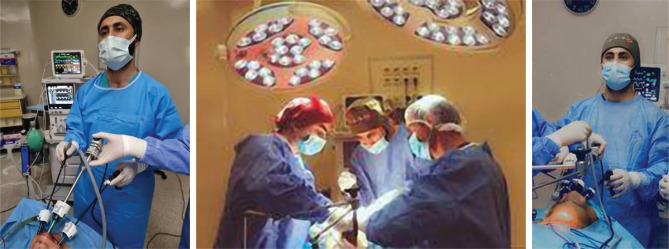
Position of the surgeon and the two assistants.

The surgeon stood at the center behind the patient's head while the two assistants stood on the right and left side of the patient's neck (Figure 2).

**Figure 3:**
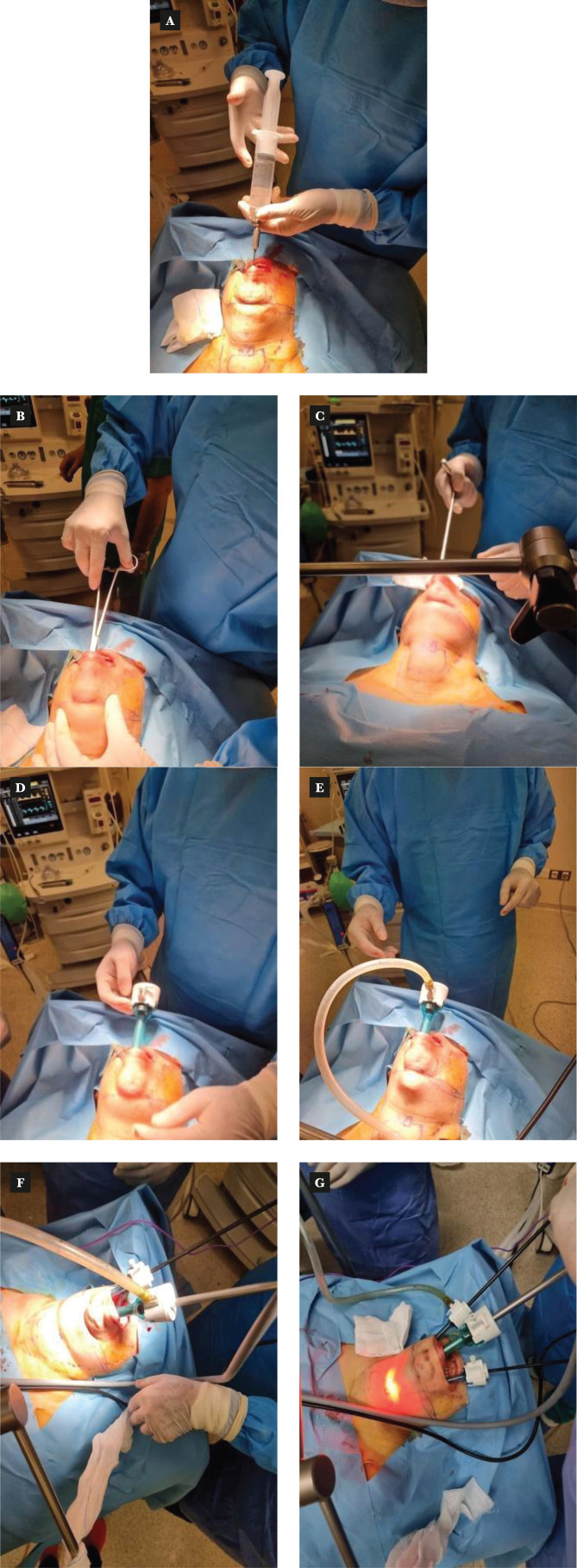
The process of hydrodissection and the insertion of all three ports with instruments. A – hydrodissection; B – kelly clamp to widen the space; C – introducing a blunt dilator to create subplatysmal space; D – inserting the central port for camera; E – CO_2_ insufflation through the central port; F – first 5 mm port inserted in the right side to do some dissection before inserting the last port; G – fine 5 mm port inserted in the left side.

A 10 mm incision was made in the center of the vestibule above the inferior labial frenulum. The central incision was dissected through the mentalis muscle down to the tip of the chin using electrocautery. Then, 1:500,000 adrenaline-saline solution was used for hydrodissection down to the anterior neck area. With the help of hydrodissection and using a Kelly clamp and a rigid blunt dilator/dissector, we approached the correct plane between the platysma and the strap muscles. Then, the blunt dissector advanced in all directions to widen the space. Then, a 10 mm trocar was inserted, and CO_2_ insufflated through it and maintained at 6 mmHg. Two 5 mm ports were inserted lateral to the canine teeth on the lower lip parallel to the 10 mm port (Figure 3 A–G).

**Figure 4:**
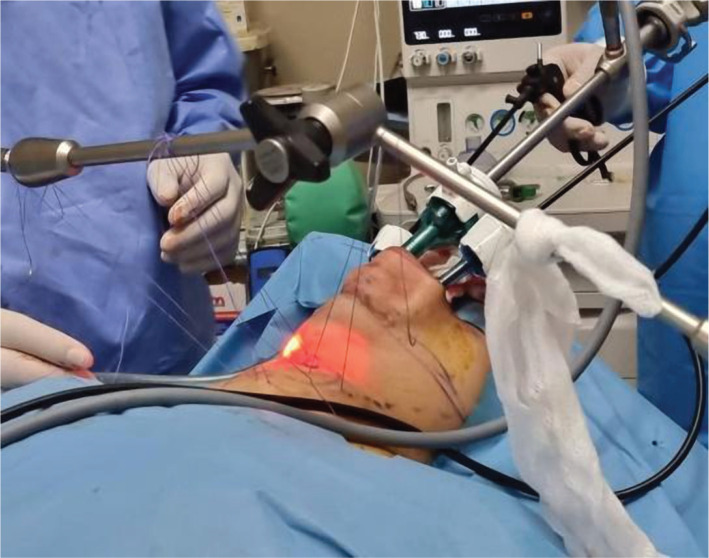
Sutures to elevate the skin and the muscles fixed on the external retractor.

After inserting the camera in the central port, monopolar cautery, ligasure device and other endoscopic dissectors and grasper were inserted in the lateral ports alternatively to dissect our working space to the end boundaries necessary. The median raphe was opened, and a 2/0 suture material was used to elevate the strap muscles laterally. An external retractor was used to fix the suture material on it (this has not been described in previous studies) (Figure 4).

**Figure 5:**
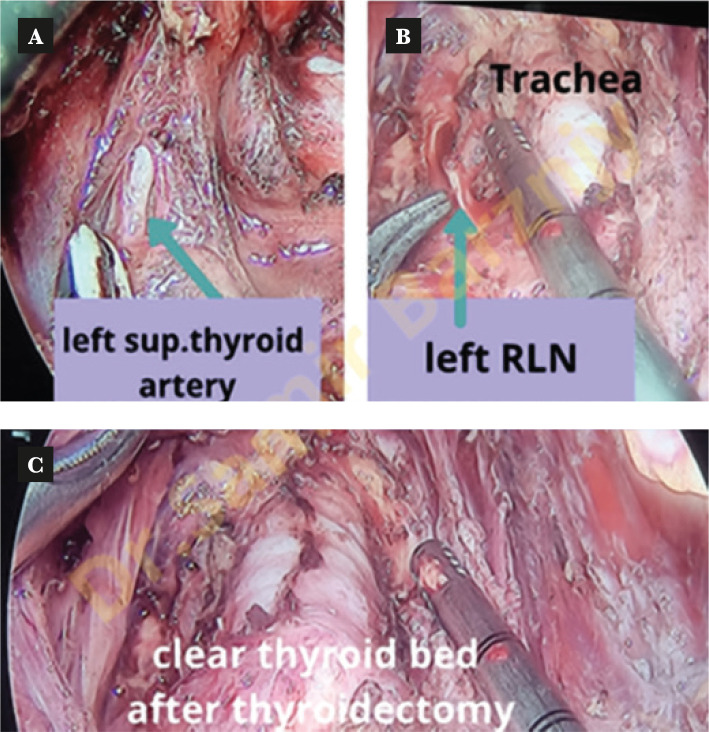
Left superior artery, RLN and trachea after the removal of the gland. A – Shows left superior thyroid artery; B – shows left RLN with trachea after the removal of the gland; C – Shows clear thyroid bed after thyroidectomy.

**Figure 6:**
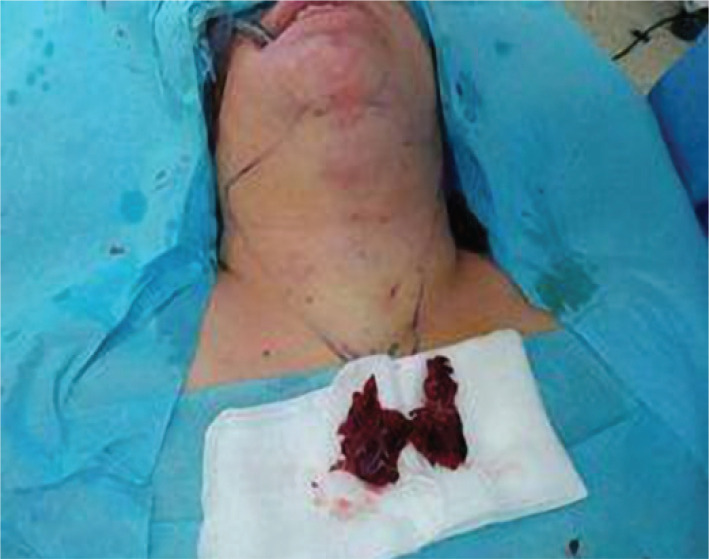
Patient after surgery following gland extraction.

The dissection of the thyroid gland was started by elevating the isthmus and dividing it. The next step was to open the Joll's space to identify and cut the superior thyroid artery. Then medial retraction of the lobe was necessary to expose the recurrent laryngeal nerve (RLN). Dissection of the gland was continued to the end carefully to preserve the RLN and the parathyroid glands (Figure 5). We made an immediate postoperative picture after the extraction of the gland (Figure 6).

Then the specimens were extracted through the 10 mm incision, and large lobes or nodules were cut once or more to ease their extraction. After finishing the procedures, the strap muscles were approximated, and the incisions were closed. We did not place surgical drains for lobectomy or total thyroidectomy cases. A pressure dressing was placed around the chin for one day. Postoperative antibiotics were prescribed for the patients for five to seven days to prevent infection.

## Results

Twelve patients underwent surgeries between November 2020 and April 2021, including eleven females and one male. Eleven total thyroidectomies and one lobectomy were done. The youngest patient was 23 years old, and the oldest patient was 50 years. The clinical data regarding the age of the patients, the volume of the gland, the size of the nodules, fine needle aspiration cytology, type of surgery, duration of surgery and time of discharge is shown in Table 1. The longest duration of surgery recorded for total thyroidectomy was 210 minutes, and the shortest was 150 minutes. We spent 130 minutes on one case of lobectomy. We had no conversions to open surgery, no recurrent laryngeal nerve injury and no hypocalcaemia. One case experienced dyspnea 5 hours after the surgery: in the first hours, she was doing well with no dyspnea, and her voice was good, then she developed dyspnea which was explained as a case of laryngeal edema and resolved after 24 hours with supportive treatments including oxygen, steroid and reassurance with no permanent complications. No drains were used for the patients, and apart from minor redness or bruising, like in other surgical wounds, no major complications were noted.

**Table 1:** Characteristics of patients who underwent TOETVA.

Patient	Gender	Age in years	Thyroid Volume in ml		Largest Nodule Size	FNAC/Bethesda	Diagnosis	Type of Surgery	Duration of Surgery	Drain	Post op discharge	Complications
			Rt Lobe	Lt Lobe								
**1**	M	37	16	9	8 mm	N/A	Toxic MNG	TT	180 min	No	18 hours	Nil
**2**	F	42	25	10	50 mm	N/A	Euthyroid MNG	TT	180 min	No	20 hours	Mild redness subsided in 3 days
**3**	F	50	15	20	35.5 mm	III	Subclinical hyperthyroidism+MNG	TT	210 min	No	20 hours	Mild swelling subsided in a few days
**4**	F	47	8	10	10 mm	III	Toxic MNG	TT	185 min	No	72 hours	Laryngeal edema, subsided in 24 hours
**5**	F	45	7	11	15 mm	II	Toxic MNG	TT	190 min	No	20 hours	Mild edema, resolved in a few days
**6**	F	31	28	12	48 mm	N/A	Euthyroid MNG	TT	150 min	No	20 hours	Nil
**7**	F	28	8	20	40 mm	II	Large complex nodule	Lobectomy	130 min	No	20 hours	Nil
8*	F	37	28	24.6	20 mm	II	Toxic MNG	TT	200 min	No	19 hours	Mild bruise and redness, subsided in 4 days
**9**	F	23	8	13	9 mm	II	Graves disease with nodules	TT	200 min	No	20 hours	Very mild bruise resolved in 2 days
**10**	F	30	9	13	No nodules	N/A	Graves disease	TT	180 min	No	20 hours	Nil
**11**	F	50	6.4	6.2	9 mm	III	Suspicious toxic nodules	TT	185 min	No	20 hours	Infection, incision and drainage were performed, completely healed with no further complications
**12**	F	39	8	9	10 mm	N/A	Toxic MNG	TT	180 min	No	20 hours	Mild non-significant bruise

The size of case number 8 was 52.6 ml which was slightly larger than the upper limit. MNG – multinodular.

Although we prescribed postoperative antibiotics for all the patients who underwent TOETVA, one case developed an infection and abscess one week after the procedure. Aspiration under antibiotic cover was not successful in treating the condition, so we decided to drain the pus under general anesthesia with a small incision about 2 cm in the upper part of the neck, which resolved the condition with no complications left behind.

**Figure 7:**
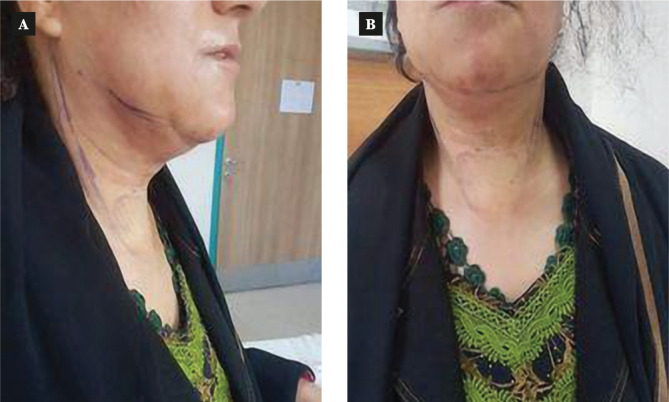
Post-operative view of the neck. A – Lateral view of the neck; B – Anterior view of the neck.

**Figure 8:**
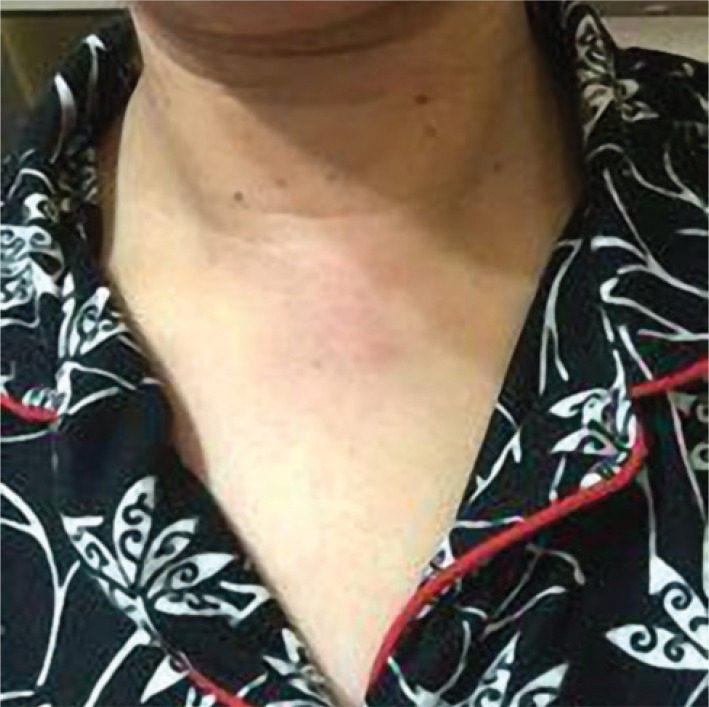
Picture of a patient on the first postoperative day.

Two pictures of the first postoperative day of a patient show anterior and lateral views of the neck (Figure 7 A–B), and one picture of another patient from the first postoperative day (Figure 8).

**Figure 9:**
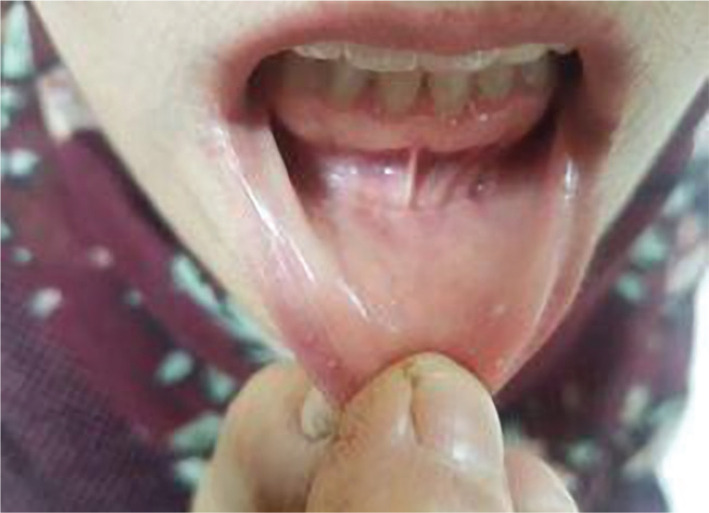
Post-operative picture of intra-oral incision.

Three weeks postoperative picture of intra-oral incision of a patient which shows complete healing of the vestibular scar (Figure 9).

## Discussion

Since the first endoscopic parathyroidectomy done by Gagner in 1996 [16], different methods have been developed for endoscopic thyroidectomy [17–21]. In general, the indications for TOETVA are benign thyroid diseases, papillary microcarcinoma and thyroid tumor size of 6 cm or less [22–24]. Apart from benign conditions, TOETVA can be done safely for patients with thyroid cancer and central neck dissection [25].

Cosmetic issues have entirely been eliminated by TOETVA. Compared to other endoscopic methods, it has some advantages, like smaller dissections and access to the right and left lobes of the thyroid gland through the same incision [26].

After transoral thyroidectomy, minor skin injuries are not uncommon. Chin and anterior neck skin injury and ecchymosis are frequently encountered in the immediate postoperative period [27, 28]. We noticed bruises and/or ecchymosis very clearly in five cases in our series.

Despite not having concomitant intraoral infections like tooth abscesses or other infectious conditions, transoral thyroidectomy is considered a clean-contaminated case as it violates the oral mucosa [29]. Adequate coverage can be provided using antibiotics like amoxicillin-sulbactam or cefazolin + an anaerobic covering antibiotic (clindamycin or metronidazole) [30]. As per the published data for TOETVA and in a general context, infections are sporadic, and major complications do not reach 5% in the published articles [3, 27]. In terms of complications and results, several authors agree that TOETVA is comparable to the classic method in selected patients [31, 32].

High complication rates and violation of mental nerve in terms of injury have been documented in previous reports [27, 33, 34]. In contrast to previous studies, a very low rate of transient nerve injury (1.5%) is documented by some authors, with complete recovery within 2–4 months. Therefore, the authors modified the position of the two lateral small ports, making the ports very lateral to the canine teeth and closer to the lower lip. So, there was no mental nerve injury after modifying the position of the lateral ports. A low incidence of RLN injury was recorded with the advances of the TOETVA procedure, and full recovery of the nerve function was reported within 6 months of surgery in all reported cases [3, 31, 35]. In our series of these twelve cases, we had no RLN injury.

The risk of mental nerve injury is one of the concerns among the authors, but it seems to be no real risk of nerve injury with the most recently described technique [36], and we did not have any cases of mental nerve injury in our first series.

A total of 82 patients were included in a study published by Mi Ra Kim et al., in which 44 cases underwent TOETVA and 38 classic open methods. No significant drop in pitch was noted in TOETVA cases [37].

TOETVA is not an easy surgery, and it is challenging even for experienced surgeons, with a steep learning curve, and it should not be undertaken by non-expert thyroid surgeons [3]. One should have several skills to perform this procedure, including advanced laparoscopy skills and working within the narrow space in the neck [3]. According to the literature, to be familiar with this technique and to ensure the needed skills to perform this novel procedure, a surgeon requires cadaveric dissections, case observations and mentored initiation of the first cases [3].

The most challenging and difficult part of the surgery for an inexperienced surgeon is the dissection of the superior pole because it is poorly visible, as, during the procedure, we see the thyroid gland craniocaudal [37]. Apart from this, in our initial experience, we noticed that insertion of the first lateral port was somewhat difficult. However, after performing several cases, it became easier (the second lateral port was easier than the first one because we did some dissections of the subplatysmal space with ligasure after CO_2_ insufflation and insertion of the first lateral port).

Two other issues should be mentioned, which are the cost and the time of the surgery. Both of them are higher in TOETVA in comparison to the classic open method; this has been mentioned by other authors as well. We noticed the long surgical time in our cases, but it was not longer than the surgical time published by many other authors [11], and it is expected to decrease after performing twenty cases [38].

Permanent hypoparathyroidism has not been reported in TOETVA [15], and fortunately, we did not have any cases of hypoparathyroidism in our series. Very few hematomas were addressed in the studies, but this may be due to the limited cases in the literature, which underestimate this complication [9]. Although the use of drains is still under discussion, we did not insert drains in all our cases and did not have any complications such as seroma or hematoma.

We did not observe major complications, such as recurrent laryngeal nerve injury, definitive hypoparathyroidism and hematoma, nor the need to convert to the cervical approach. Although postoperative antibiotics were given to all the patients who underwent this procedure, we had one case of infection and abscess formation, which did not resolve with aspiration under antibiotic cover. Therefore, we decided to make a small incision and drain the pus under sedation, and the patient completely recovered after one week with no other complications.

There are articles published worldwide detailing the safety and effectiveness of TOETVA; therefore, this procedure should no longer be regarded as an experimental operation [9].

## Conclusion

We noticed that TOETVA is safe and feasible, nearly with the same complications as the classic open method but completely avoiding the neck scar. Operation time is long, but it decreases with experience. It needs a good knowledge of the neck and thyroid anatomy, training and very good laparoscopic skills to perform this surgery, and it should be performed cautiously. Our initial experience is completely positive for this amazing technique of thyroidectomy. We will publish more articles with a larger number of cases in the future.
